# Shining a Light on the Future of Biophotonics

**DOI:** 10.1002/jbio.202500148

**Published:** 2025-05-14

**Authors:** Francesco Baldini, Kishan Dholakia, Paul French, Orlando Guntinas‐Lichius, Achim Kohler, Werner Mäntele, Laura Marcu, Ronald Sroka, Siva Umapathy, Juergen Popp

**Affiliations:** ^1^ Institute of Applied Physics “Nello Carrara” (IFAC), National Research Council of Italy (CNR) Sesto Fiorentino Italy; ^2^ Centre of Light for Life & Institute for Photonics and Advanced Sensing & School of Biological Sciences University of Adelaide Adelaide South Australia Australia; ^3^ SUPA, School of Physics & Astronomy University of St Andrews St Andrews UK; ^4^ LIGHT Community, Physics Department Imperial College London UK; ^5^ The Francis Crick Institute London UK; ^6^ Department of Otorhinolaryngology Jena University Hospital Jena Germany; ^7^ Facial‐Nerve‐Center Jena Jena University Hospital Jena Germany; ^8^ Center of Rare Diseases Jena Jena University Hospital Jena Germany; ^9^ Faculty of Science and Technology Norwegian University of Life Sciences Ås Norway; ^10^ Institute of Biophysics, Goethe University Frankfurt Frankfurt am Main Germany; ^11^ DiaMonTech AG Berlin Germany; ^12^ Department of Biomedical Engineering University of California Davis California USA; ^13^ Department of Neurological Surgery University of California Davis California USA; ^14^ Laser‐Forschungslabor, LIFE Center, University Hospital, Ludwig‐Maximilian University Planegg Germany; ^15^ German Society for Biophotonics and Laser Medicine, c/o Evangelische Elisabeth Klinik, Zentrum Lasermedizin Berlin Germany; ^16^ Department of Inorganic and Physical Chemistry Indian Institute of Science Bangalore India; ^17^ Department of Instrumentation and Applied Physics Indian Institute of Science Bangalore India; ^18^ Leibniz Institute of Photonic Technology (IPHT) Jena Germany; ^19^ Institute of Physical Chemistry and Abbe Center of Photonics, Friedrich Schiller University Jena Germany

**Keywords:** artificial intelligence, bioimaging, biophotonics, biosensing, clinical translation, laser medicine, one health, optical health technologies, photonic therapy

## Abstract

Biophotonics—the interdisciplinary fusion of light‐based technologies with biology and medicine—is rapidly transforming research, diagnostics, and therapy across various domains. This white paper, developed in conjunction with the International Congress on Biophotonics 2024, offers a comprehensive overview of the current landscape and future potential of biophotonics. It discusses core technologies such as bioimaging, biosensing, and photonic‐based therapies, while highlighting novel applications in oncology, infectious diseases, neurology, cardiovascular health, agriculture, food safety, and environmental monitoring. The document also explores key enablers, including artificial intelligence, novel materials, and quantum biophotonics, along with critical challenges related to standardization, regulation, and clinical translation. A SWOT analysis and recommendations are provided to guide future research, commercialization, and interdisciplinary collaboration, underscoring biophotonics as a cornerstone of next‐generation precision medicine and the One Health approach.

## 1 Introduction

Biophotonics, the innovative convergence of biology, medicine, artificial intelligence (AI), and photonics, represents a transformative force in numerous scientific and medical fields. This dynamic discipline employs the use of light to analyze and manipulate biological materials, thereby offering new opportunities for precision measurements in the fields of fundamental and applied research, medical diagnostics, and treatment, including laser medicine. The potential applications of biophotonics are numerous and diverse, encompassing fundamental investigations of cell processes as well as health‐related applications such as diagnostics, monitoring therapy, and well‐being. Additionally, biophotonic technologies have the potential to contribute to environmental and food monitoring and agricultural advancements. This article aims to present a comprehensive exploration of the critical discussions and insights shared during the “International Congress on Biophotonics” held in March 2024 in Jena. This congress brought together leading experts, researchers, and practitioners who explored the transformative capabilities of biophotonics, addressing both its current applications and future prospects. As we examine the future of biophotonics, this article aims to highlight the significant strides made in the field and the promising horizons yet to be explored.

## 2 Biophotonics: A Short Definition and Overview

Photonics encompasses the generation, control, manipulation, propagation, measurement, and utilization of light. Recognized as a key technology of the 21st century, photonic technologies play a crucial role in addressing significant societal challenges and driving advancements in areas like life sciences, communication, and production.

**FIGURE 1:**
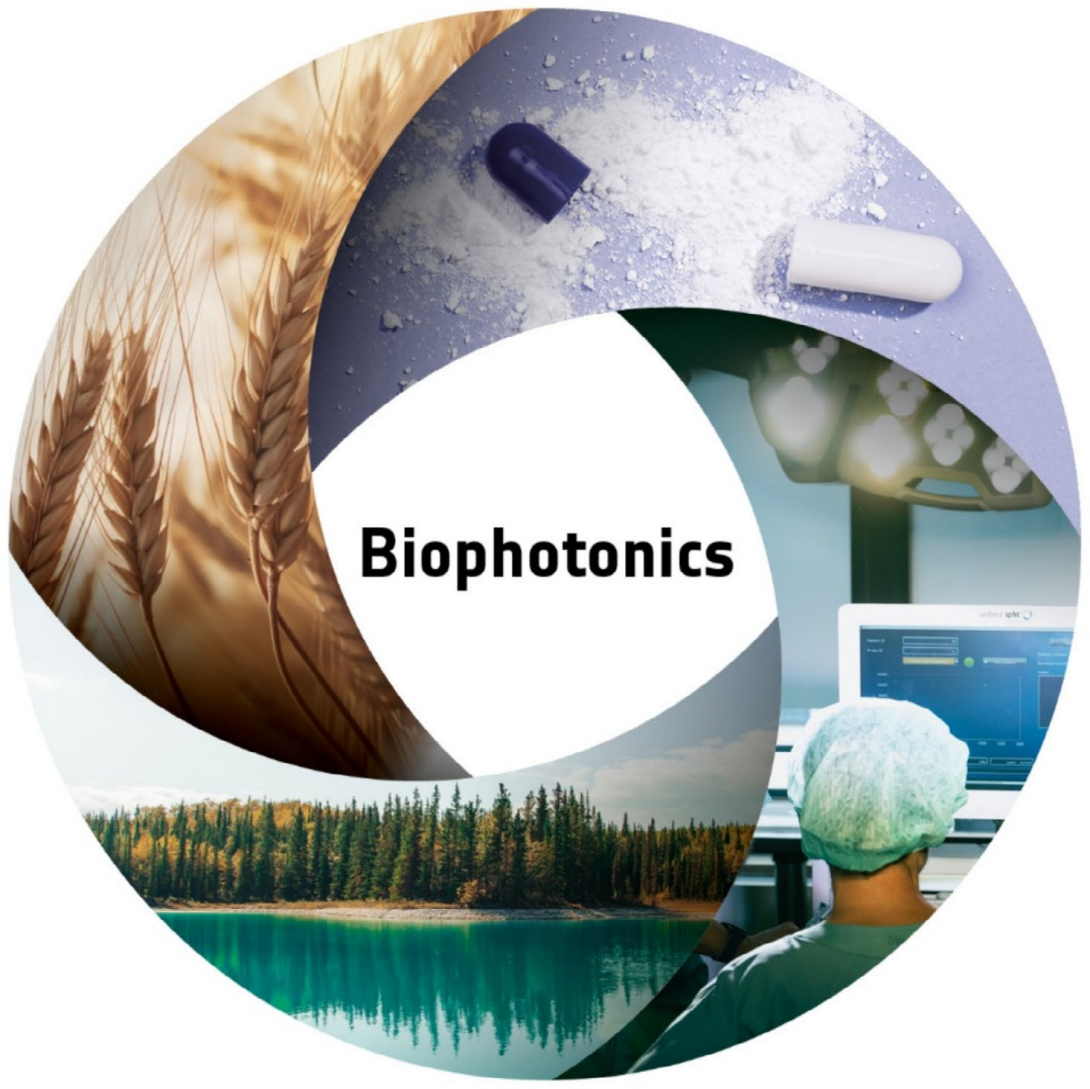
Areas of biophotonics in the spirit of the One Health approach.

Biophotonics is a rapidly advancing field at the intersection of light and biological systems, representing a significant leap forward in natural sciences, but also medicine. The term “biophotonics” combines the Greek words “bios” (life) and “phos” (light), reflecting its core focus: exploring how light interacts with biological matter. By leveraging photonics—the science and technology of manipulating light—biophotonics has emerged as a powerful tool for understanding and influencing life processes at the molecular, cellular, tissue, and organ levels. The field seeks to understand biological processes through optical methods, drawing inspiration from nature—such as the way plants harness light through photosynthesis or the complex processes that occur in human vision. The journey to understanding light, from early antiquity to Albert Einstein's photon theory, parallels the challenge of comprehending life, studied from Aristotle to modern genomics (Figures 1).

**FIGURE 2:**
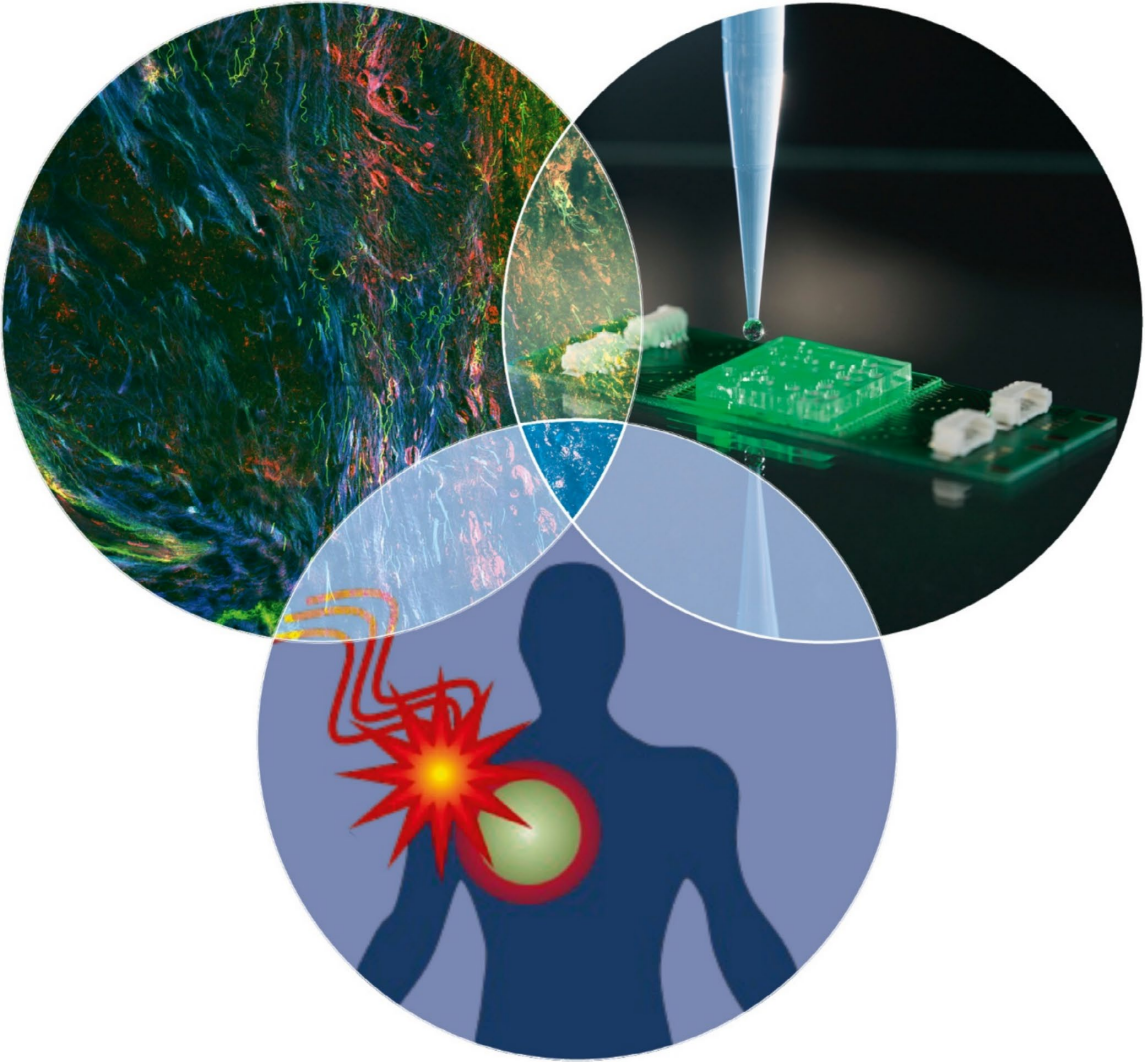
The three main areas of biophotonics in health: Bioimaging, biosensing, and treatment control.

Biophotonics is not merely an academic pursuit; it offers groundbreaking possibilities for both fundamental research and practical applications across various industries, including pharmaceuticals, food, biotechnology, and most importantly medicine. Innovative optical techniques developed through biophotonics allow scientists to capture cellular conditions and monitor dynamic processes, providing a comprehensive view of life processes within biological systems (e.g., at molecular levels, in cells, in tissues, and in organs). Biophotonic techniques involve studying the structural, functional, mechanical, biological, and chemical properties of biological materials through light interactions such as absorption, emission, reflection, and scattering. In addition, lasers and other advanced light sources are applied for surgery and treatment. Given the vastness of the field, we refrain from citing original literature and instead provide a selection of comprehensive textbooks at the end of the text to help interested readers gain an overview [1, 2, 3, 4, 5, 6, 7] (Figures 2).

Some key advantages of using light in biophotonics include:

- Non‐contact measurement: The utilization of light facilitates the observation of living cells in a noninvasive manner, thereby ensuring the preservation of the integrity of the analyzed cells and the absence of any toxic effects.
- Speed and instant information: Optical measurements have the capacity to provide rapid, real‐time data, thereby significantly reducing the time required for data interpretation and diagnosis.
- Sensitivity: Optical technologies allow for ultrasensitive detection, down to single molecules, which is essential to understand fundamental biological processes.
- Time resolution: Optical methods allow the observation of dynamic biological processes over a range of temporal scales, from hours to ultrafast reactions.

One important goal of biophotonics is to unravel mechanisms behind the origins of diseases, enabling prevention, early diagnosis, and targeted or personalized treatment and follow‐up treatment. While the primary focus of biophotonics is on biology research and healthcare—promising early and accurate diagnosis and efficient treatment (including treatment with light)—it also extends to environmental monitoring and agricultural advancements.

Achieving these ambitious goals requires interdisciplinary collaboration. Physicists, chemists, engineers, biologists, computer scientists, medical professionals, and industry stakeholders must work together to fully realize the potential of biophotonics. This cooperation is essential in transforming innovative observations made in the lab into diagnostic and treatment methods for patients addressing unmet medical needs.

In summary, biophotonics stands at the forefront of scientific innovation, offering profound insights into biological or biomedical processes and paving the way for new diagnostic and therapeutic approaches. As the field continues to evolve, its impact on both science and society is poised to grow, making it a vital area of research and application in the 21st century.

Before we come to the actual core of this manuscript, a critical review of the findings of the “International Congress on Biophotonics” in March 2024 (ICOB 2024), we would like to give a brief summary of the core topic of biophotonic research on the interaction of light‐biological matter, which is the basis of the most important biophotonic approaches that were also the subject of the ICOB 2024.

### 2.1 Biophotonics: A Short Summary of the Key Technologies

The field of biophotonics can be divided into the following main areas:

- *Bioimaging*: Photonics technologies enable the characterization of biological specimens across multiple spatial scales, from the nanoscopic level—facilitating the investigation of intracellular interactions—to the microscopic and macroscopic domains, including tissues and muscle structures.
- *Biosensing*: Photonic‐based approaches allow for the detection of biomolecules, such as disease‐specific biomarkers, with sensitivities reaching molecular concentrations and, in principle, single‐molecule resolution.
- *Treatment and treatment control*: Lasers and other light sources are in use for facilitating highly precise and minimally invasive surgical interventions, while bioimaging and biosensing modalities enable real‐time monitoring of treatment efficacy and post‐operative recovery biosensing.

These three areas can efficiently run separately, but they can work in parallel, offering even greater potential.

#### 2.1.1 Bioimaging

The majority of currently available biophotonic techniques can be explained in terms of light‐biological matter interaction. The interaction phenomena are predominantly based on the processes of absorption, emission, scattering, and reflection. These phenomena are distinguished by their capacity to elucidate a vast array of morphological and molecular intricacies across a spectrum of size scales, encompassing macroscopic, microscopic, and nanoscopic resolutions.

The most representative label‐free biophotonic diagnostic methods include hyperspectral imaging (HSI), fluorescence imaging and fluorescence lifetime imaging (FLIM) of endogenous fluorophores, second harmonic generation (SHG), third harmonic generation (THG), optical coherence tomography (OCT), diffuse remission spectroscopy (DRS), photoacoustic imaging (PAI), vibrational microspectroscopy (infrared [IR] absorption and Raman scattering), and Brillouin scattering. The contrast mechanisms underlying these methods can be highly molecule‐specific in the case of IR absorption and Raman scattering; it can visualize native electronic chromophores such as hemoglobin, NADP(H), flavin, elastin, or cytochrome by absorption (HSI or PAI) or emission (autofluorescence or fluorescence label imaging); or it can visualize specific structural proteins (e.g., collagen) by SHG as well as changes in refractive index (OCT) or phase boundaries (THG).

Molecular contrast is highest when spectroscopic data are acquired, as in HSI, FLIM, spontaneous or coherent Raman spectroscopy, or IR absorption spectroscopy. These techniques enable visualization of the spatial distribution of molecular markers such as proteins, lipids, or DNA. In contrast, methods such as OCT detect changes in refractive index, which are often not directly correlated with specific molecular structures but provide detailed imaging of tissue architecture down to the cellular level. Conventional OCT can be extended to spectroscopic OCT (SOCT), which provides localized spectroscopic information based on the principles of OCT. SOCT captures the spectral content of backscattered light, enabling the quantification of depth‐resolved spectra to determine the concentration of tissue chromophores (e.g., hemoglobin and bilirubin). However, acquiring spectral information is time‐consuming, so such methods are generally slower than methods that measure only a single parameter per pixel, such as normal OCT. OCT is currently one of the fastest methods in terms of volume elements (voxels) imaged per second, enabling real‐time 3D imaging of dynamic processes, and is already widely established in ophthalmology.

It is useful to distinguish between linear and nonlinear light‐matter interaction phenomena. Recent advances in the development of compact and easy‐to‐operate high‐intensity ultrashort laser sources enabled the exploitation of nonlinear optical phenomena for biomedical imaging, resulting in significant improvements in penetration depth, optical resolution as well as acquisition speed. In particular, multi‐photon absorption is especially valuable for microscopy applications. Simultaneous absorption of two or three photons leads to precise localization of the sources of fluorescence or SHG and THG signals, since such nonlinear processes can only take place in an extremely small volume. Multi‐photon imaging using near IR (NIR) femtosecond lasers can provide high penetration depths and therefore allows the study of biological tissue with high spatial resolution and good contrast not only on the surface but also deep within the tissue.

The disadvantage of linear Raman spectroscopy is the intrinsically small scattering cross‐sections, which make it impossible to acquire hyperspectral Raman images of larger tissue areas. This can be overcome by using nonlinear coherent Raman scattering (CRS) phenomena such as CARS (coherent anti‐Stokes Raman scattering) and SRS (stimulated Raman scattering). CARS and SRS enhance the intrinsically weak Raman signal and avoid being swamped by autofluorescence background, but they are reduced in molecular selectivity because they can image only one or a few characteristic Raman bands and are more complex than standard Raman approaches.

Whereas the spatial resolution of the aforementioned optical imaging methods is usually limited by the diffraction limit, novel fluorescence‐based nanoscopy techniques such as STED, PALM/STORM, SIM, Airyscan, and MINFLUX enable us to go below the diffraction limit by utilizing optical fluorescence transitions. However, these super‐resolution fluorescence approaches are not label‐free and require environmental‐sensitive fluorescence probes. Such fluorescence nanoscopy techniques enable highly sensitive observation of subtle changes in cellular properties, such as membrane fluidity and integrity, particularly in response to inflammation. Moreover, novel approaches like adaptive optics or endoscopy allow for deeper imaging within tissue.

**FIGURE 3:**
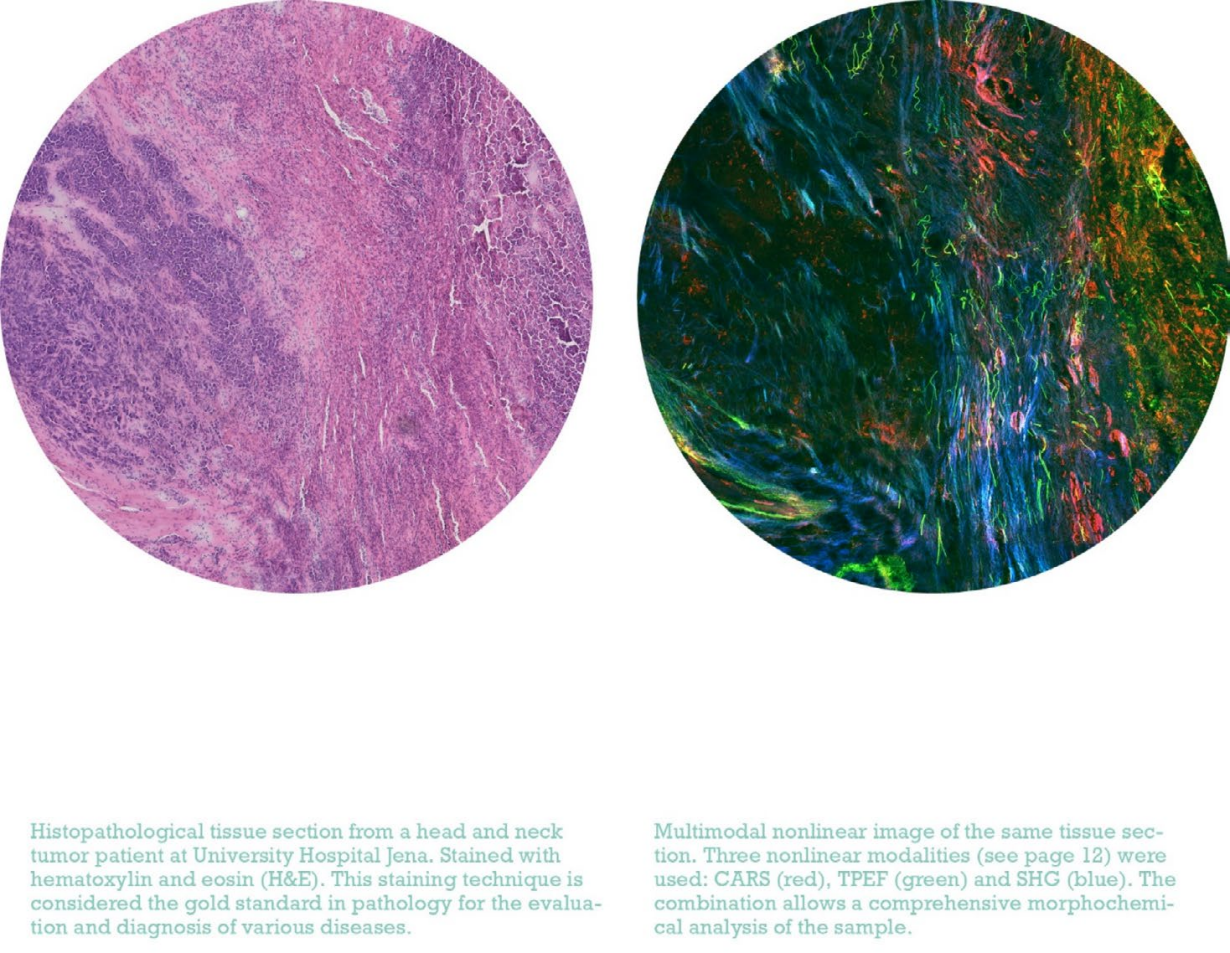
Comparison of an H&E‐stained image of a tissue section of a tumor patient and a multimodal image based on various nonlinear modalities (CARS, red; TPEF, green; SHG, blue).

To extend the potential of all these optical approaches, it has proven to be advantageous to combine several contrast mechanisms in a multimodal approach. Multi‐contrast imaging has become a powerful tool of biomedical research. One can think of two end‐member strategies for combining these diverse methods. On one end, imaging modalities with similar acquisition speed and resolution can be combined, for example, nonlinear imaging methods like Multi‐Photon Excited Fluorescence (MPEF), SHG, and CARS, all of which are efficiently excited by the same laser source and can be detected in parallel. On the other end, imaging modalities of different imaging speeds and tissue penetration can be synergistically combined, so that a fast but chemically less specific method provides an overview of the tissue volume or larger area, while a slower, molecule‐specific second method is used to classify tissues detected by the faster modality in suspicious but small areas. One such approach would be to combine OCT or FLIM with Raman (Figure 3).

#### 2.1.2 Biosensing

Unlike bioimaging, where most approaches are label‐free, biosensing employs a more balanced approach, incorporating both label‐free and label‐based methods to detect and, when possible, to quantify specific biomolecules.

##### 2.1.2.1 Label‐Free Approach

Biomolecules can be detected using either their intrinsic optical properties (fluorescence, absorption, and Raman scattering) or through an indirect measurement of refractive index changes induced by the biomolecular interaction between the biomolecule and a biological recognition element (antibodies, enzymes, nucleic acids, aptamers, among others).

In the initial case, for instance, the presence of bile within the gastroesophageal tract can be identified through the measurement of bilirubin absorption within the gastroesophageal content. The utilization of optical fiber catheters, culminating in a mini‐spectrophotometric cell that is open to the external environment, facilitates uninterrupted, 24‐h monitoring of bile‐containing refluxes. In Raman scattering, the performances characterized by an intrinsically weak cross‐section can be greatly improved by developing sensing probes using metallic surfaces, mainly gold, and silver, as substrates for the biosensing layer interacting with specific biomolecules. With this approach, the so‐called surface‐enhanced Raman scattering, the huge local enhancement of the electromagnetic field over nanometric distances from the metal surfaces leads to up to a million‐fold increase of the Raman signal leading to the achievement of very low limit of detections.

A biosensing layer containing the biological recognition element and deposited on suitable surfaces is also used when the measurement of the biomolecule concentration is carried out through a measurement of refractive index changes. In optical biosensors based on surface plasmon resonance (SPR), the biosensing layer is deposited on gold surfaces and the interaction between optical waves and propagating surface plasmons occurs on macroscopic surfaces with plasmon propagation lengths in the micron range. The condition of coupling between the optical wave and the surface plasmon is modulated by the change of the refractive index of the biosensing layer and the shift in the resonance peak becomes dependent on the concentration of the biomolecules “captured” by the biosensing layer. In localized surface plasmon resonance (LSPR), gold nanostructures, such as nanoparticles, or array of nanoobjects on suitable substrates, are used: the light interaction is with localized surface plasmons that undergo collective harmonic oscillations under an applied electric field with the advantage that the excitation is performed with freely propagating light and does not require the special coupling geometries used in SPR such as prism coupling, grating coupling or waveguide coupling.

Another label‐free approach exploits the use of optical resonators as extremely sensitive structures capable to go down to single molecule detection thanks to the incredibly high interaction of the so‐called whispering gallery modes (WGMs) propagating within optical cavities with the biosensing layer deposited on their surface. From the first application to bulk optical cavities such as glass microspheres, microrings, and microtoroids up to the optofluidic resonators as microbubbles, these structures can be characterized by unreachable performances even if excitation of WGM is often reached with not simple optical geometries.

Optical waveguides are used as label‐free optical platforms for biosensing when the interactions with the biosensing layer occur through the cladding modes of the evanescent field that extends around the waveguide core in the fiber cladding. The interactions of the biomolecule with the biosensing layer induce changes in the refractive index surrounding the core that modulate the resonance peak of the cladding modes as a function of the biomolecule concentration. In the context of optical fiber waveguide biosensing, the most prevalent strategies encompass long‐period gratings, tilted fiber Bragg gratings, and fibers coated with a thin layer of metal oxide, which collectively facilitates lossy mode resonance.

##### 2.1.2.2 Label‐Based Approach

Fluorescence labels are widely used in in vitro biosensing for the detection and quantification of biomolecules, providing high sensitivity and specificity. These labels are fluorophores—molecules that absorb light at a specific wavelength and emit light at a longer wavelength—allowing researchers to track biological interactions in real time.

Common applications of fluorescence labeling in biosensing include immunoassays, nucleic acid detection, and protein analysis. Fluorescent dyes, quantum dots, and fluorescent proteins such as GFP (green fluorescent protein) serve as reliable markers in these assays. Techniques like fluorescence resonance energy transfer (FRET) and FLIM further enhance detection accuracy by providing additional information about molecular interactions and environmental conditions.

Fluorescent biosensors are extensively used in diagnostic platforms, including microarrays and lab‐on‐a‐chip devices, enabling rapid and precise disease detection. Moreover, the integration of fluorescence with advanced microscopy and flow cytometry allows for high‐throughput analysis of cellular and molecular processes.

**FIGURE 4:**
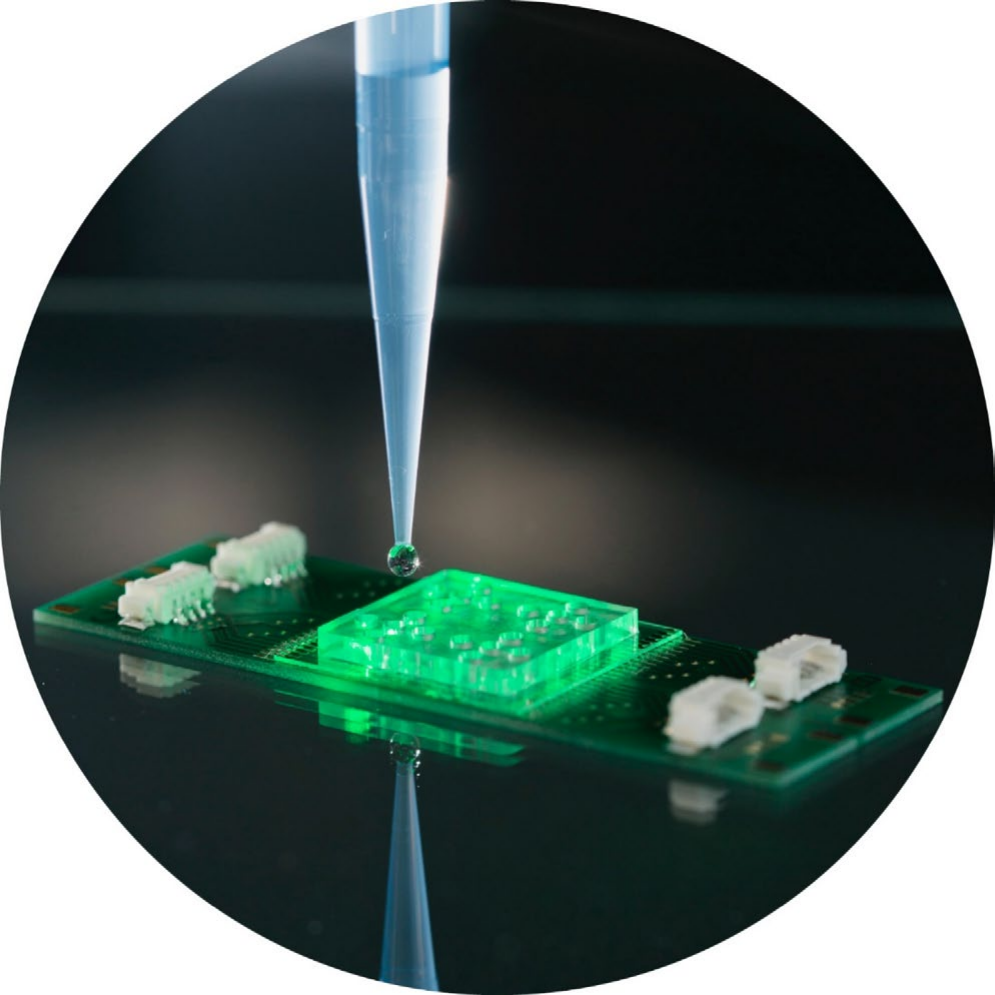
Assay with which bacteria can be identified and their resistance against certain antibiotics can be tested employing Raman spectroscopy.

Despite their advantages, challenges such as photobleaching, background noise, and nonspecific binding must be addressed to improve assay performance. Ongoing research aims to develop brighter, more stable fluorophores and innovative labeling strategies to enhance the effectiveness of fluorescence‐based biosensing in biomedical applications (Figure 4).

#### 2.1.3 Treatment and Treatment Control

Light‐based medical technologies have revolutionized modern healthcare by offering minimally invasive solutions for surgery, treatment and treatment control. Optical methods provide precise, targeted interventions, reducing patient recovery time while enhancing treatment efficacy. Advances in lasers, photodynamic therapy (PDT), and optical imaging have significantly improved patient outcomes across multiple medical fields.

Laser surgery is one of the most prominent applications of light in modern medicine. Lasers provide precise cutting, coagulation, and ablation or fragmentation of tissues with minimal collateral damage. Various wavelengths are employed depending on tissue type and desired outcomes. For example, carbon dioxide (CO_2_) lasers are commonly used for soft tissue surgeries, while Nd:YAG and diode lasers are frequently employed in ophthalmology, dermatology, and oncology.

One of the greatest advantages of laser surgery is its ability to minimize bleeding by sealing blood vessels as it cuts. This feature is particularly valuable in delicate procedures such as retinal surgery and neurosurgery. Additionally, robotic‐assisted laser surgery allows for highly accurate interventions, improving surgical precision and reducing human error.

**FIGURE 5:**
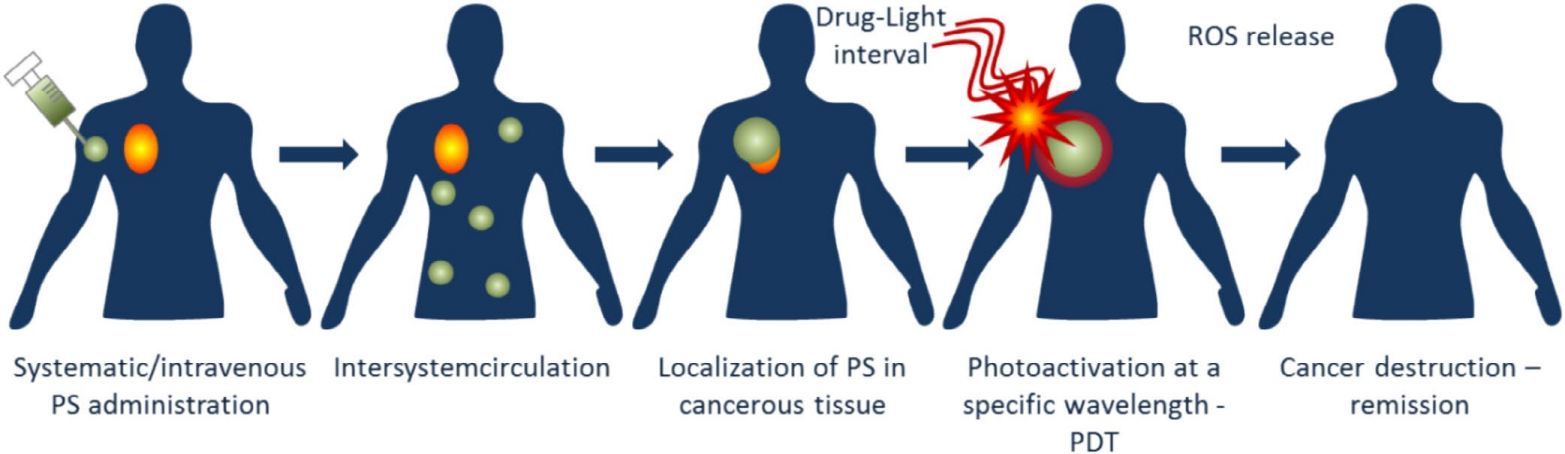
Workflow of photodynamic therapy (PDT) after photosensitizer (PS) administration (taken over from EPS Grand Challenges, CC BY‐SA 4.0; ROS stands for reactive oxygen species).

PDT is a widely used light‐based treatment, particularly in oncology and dermatology. PDT involves the administration of a photosensitizing agent that accumulates in diseased tissue. When exposed to specific wavelengths of light, the agent produces reactive oxygen species that selectively destroy abnormal cells while sparing healthy tissue. This approach has shown effectiveness in treating certain types of skin cancers, precancerous lesions, and macular degeneration (Figure 5).

In dermatology, intense pulsed light and laser therapy are used to treat conditions such as acne, vascular lesions, and pigmentation disorders. Similarly, low‐level laser therapy, also known as photobiomodulation, promotes tissue repair and reduces inflammation in conditions ranging from musculoskeletal injuries to chronic pain disorders.

Furthermore, laser‐based techniques are increasingly employed in dentistry, where they facilitate cavity removal, gum disease treatment, and teeth whitening with reduced discomfort and improved precision.

Optical imaging and sensor technologies as described above play a crucial role in monitoring treatment progress and ensuring therapeutic efficacy.

Biosensing and bioimaging can be combined together offering outstanding performances as in the case of intracellular nanosensing with optical switches able to emit a fluorescent signal as a consequence of the interaction with intracellular biomolecules; for example, with this approach biomolecules such as RNA messengers can be detected in the cytoplasm and at the same time followed in their intracellular pathway; molecular switches can also be engineered to interact with specific portions of the RNA messengers blocking definite functionalities and acting as theranostic agents.

Light‐based medical technologies have profoundly transformed surgical procedures, therapeutic interventions, and treatment monitoring. By offering precision, minimal invasiveness, and improved patient outcomes, optical methods continue to advance medicine across diverse disciplines. As research progresses, emerging innovations will further enhance the capabilities of light‐based technologies, paving the way for safer and more effective treatments in the future.

### 2.2 International Congress on Biophotonics 2024—A Hub for International Experts in Optical Health Technologies

From March 3 to 7, 2024, Jena served as a focal point for international experts in the field of biophotonics, with a particular emphasis on optical health technologies. The seventh edition of the International Congress on Biophotonics (ICOB 2024) brought together leading minds from science, technology, and medicine with representatives from the industry to discuss the future direction of this research field and to advance the implementation of light‐based technologies in clinical applications. The congress was organized by the Jena Leibniz Institute of Photonic Technology (Leibniz‐IPHT).

The event kicked off with Chemistry Nobel Laureate Stefan Hell on March 4. His pioneering work on light microscopy redefined the limits of spatial resolution, enabling profound insights into the nano‐world of living cells. This discovery opened new paths for biomedical research, from studying neurodegenerative diseases to exploring the mechanisms of cancer cells.

The presentations at the ICOB 2024 emphasized the interdisciplinary nature of biophotonics as a pivotal field in the development of novel methodologies in medicine and healthcare, as well as for more precise environmental monitoring. The ICOB congress demonstrated the potential of innovative biophotonic approaches for the early detection and treatment of cancer and infectious diseases and presented successful examples of technology transfer.

Biophotonic research is typically driven by unmet needs. The most important unmet needs that can be solved with biophotonic approaches are briefly summarized below, highlighting new application areas and also technological advances, as discussed in the corresponding sessions at ICOB.

### 2.3 New Applications of Biophotonics, New Technological Trends, Challenges, and Future Directions to Address Unmet Needs

#### 2.3.1 Oncology

In the field of oncology, biophotonic applications are driven by the critical need for early and accurate detection of tumors and cancerous growth, as well as for the development of effective treatment strategies, which include image‐guided surgery and treatment monitoring. Conventional diagnostic techniques frequently prove inadequate for achieving the discrimination between tumor and normal cells and tumor margin detection in radial and depth. Moreover, there is an urgent need for the development of new methods and approaches for rapid and reliable intraoperative tissue diagnosis, as well as tumor margin detection. Label‐free biophotonic techniques, including OCT, autofluorescence microscopy utilizing both fluorescence intensity and lifetime, PAI, higher harmonics generation, and linear and non‐linear Raman spectroscopy, represent the most significant approaches in this field. They offer high‐resolution imaging at the cellular level, facilitating the early detection and precise delineation of tumors, particularly when employed in a multimodal approach. These techniques facilitate accurate differentiation between malignant and benign tissues, thereby enhancing the possibility to detect the tumor margins and reducing recurrence rates. Biophotonic methods that require labels need to rely on labels that are already approved; otherwise, labels would require approvement of safety, efficacy, and quality like drugs.

Another significant application in the field of oncology is PDT. This treatment entails the utilization of light‐sensitive pharmaceutical agents that are activated by specific wavelengths of light, thereby facilitating the selective destruction of cancer cells. PDT provides a minimally invasive alternative to conventional therapies, thereby reducing damage to surrounding healthy tissues. Unfortunately, these agents also require drug approval. Moreover, the application of AI will help to analyze spectral data of complex biological matter.

#### 2.3.2 Infectious Diseases

The field of infectious diseases is characterized by a significant unmet need for the development of rapid and precise diagnostic methods. To successfully and rapidly treat infectious diseases, three unmet needs must be addressed: (i) determination of the immune response; (ii) rapid identification of the infection‐causing pathogen and, in the case of bacterial infections, its resistance pattern; and (iii) response to treatment. Biophotonics offers solutions through techniques such as Raman spectroscopy and fluorescence‐based methods, which address these unmet needs. Biophotonic approaches can encompass the entire process chain, from sampling to the final diagnostic result. They have the potential to significantly reduce the critical parameter, namely time, to initiate a personalized, lifesaving therapy when compared to the gold standard, which is mostly culture‐based microbiology. By reducing the time required for diagnosis, biophotonics has the potential to enhance patient outcomes and contribute to the control of the spread of infectious diseases.

#### 2.3.3 Cardiovascular Diseases

Cardiovascular diseases represent a significant global burden in terms of morbidity and mortality. As with the aforementioned diseases, early detection and continuous monitoring are of vital importance for the prevention of severe events such as myocardial infarction and stroke. Conventional diagnostic techniques may be perceived as invasive and uncomfortable by patients. In this context, biophotonic techniques such as OCT and PAI can provide non‐invasive, high‐resolution imaging of blood vessels and tissues, thereby facilitating the early detection of conditions such as arteriosclerosis.

Furthermore, biophotonics plays a pivotal role in the guidance of cardiovascular and probe/catheter‐based minimally invasive surgeries. The capacity to image in real time during surgical procedures enables surgeons to accurately visualize blood flow and vessel morphology, thereby facilitating the precise placement of stents and other devices.

#### 2.3.4 Neurodegenerative Diseases

Additionally, neurodegenerative diseases such as Alzheimer's disease and Parkinson's disease pose considerable challenges with regard to early diagnosis and monitoring. These conditions are typified by insidious early symptoms and intricate pathologies that traditional imaging methods frequently prove unable to discern. Biophotonics provides researchers with powerful tools for studying these diseases at the molecular and cellular levels. Techniques such as two‐photon fluorescence microscopy and light‐sheet microscopy provide high‐resolution imaging of brain tissues, thereby enabling researchers to observe changes in neuronal structures and functions.

Optogenetics, which employs light to regulate and observe neuronal activity, has facilitated a deeper comprehension of neural circuits and their involvement in neurodegenerative disorders. This technique enables the precise manipulation of specific neurons, thereby facilitating insights into disease mechanisms and potential therapeutic targets. The development of optical clearing methods that render tissues transparent has further enhanced the ability to study deep brain structures and connectivity.

Biosensors can allow the detection of specific biomarkers in cerebrospinal fluid (e.g., tau protein, Amyloid β‐protein), the determination of which with very low limit of detection can offer the possibility of the identification of the disease at its very early stage.

#### 2.3.5 Therapeutic Drug Discovery

In order to develop new therapeutic agents and optimize existing treatments, drug monitoring discovery programs are addressing all of the above diseases. These programs routinely utilize biophotonic technologies for the assay of fundamental cell biology processes, mechanisms of action, and responses to drug candidates. Biophotonic techniques are ubiquitous in drug discovery, providing spectroscopic readouts of molecular processes in solution‐based assays and increasingly advanced automated microscopy readouts in high content analysis (HCA). Conventionally, drug discovery assays have utilized solution phase or “2D cell monolayers” because they are easily prepared for high throughput assays, transparent and highly amenable to optical techniques. However, there has been a recent surge of interest in assays utilizing more complex “3D” cell cultures, such as spheroids of cells that interact with each other and patient‐derived organoids that more accurately replicate human physiology when studying the responses of cells to drugs. The increased complexity of these 3D cell‐based disease models necessitates the development of increasingly sophisticated microscopy techniques and image data analysis pipelines.

The measurement of the concentrations of the drugs after their administration is fundamental in many clinical applications, such as pulmonary infections, critical care medicine, neurology, and psychiatry. Narrow therapeutic ranges and variable pharmacokinetics from patient to patient impose a strict and frequent monitoring of the administrated drugs and the critical role of their dosage can define the alternative between life (efficacy) and death (either toxicity or subtherapeutic exposure). For example, in the case of transplanted patients, the dosage monitoring of immunosuppressants (e.g., cyclosporin A, tacrolimus, and mycophenolic acid) is a fundamental part of solid organ post‐transplant follow‐up with high levels generating side effects and low levels increasing the risk of rejection. Therapeutic drug monitoring is traditionally based on the measurement of the trough level, defined as the plasma level of a pharmaceutical product measured just before the next dose, but, in many cases, this is not sufficient and a frequent/continuous measurement of the administrated drugs is essential to follow exactly the pharmacokinetics individually and to determine efficiently the area under the plot of plasma concentration of a drug vs. time after dosage. Traditional laboratory methods such as chromatography do not consent to follow this strategy, and optical biosensors are becoming more and more essential with their potentiality of real‐time monitoring.

#### 2.3.6 Well‐Being

The field of biophotonics has expanded to encompass a range of applications in the domain of comprehensive health monitoring and well‐being. Wearable biophotonic sensors, such as those designed for noninvasive glucose monitoring, provide continuous health data that can be utilized for the management of chronic conditions like diabetes. These sensors utilize techniques such as Raman spectroscopy, NIR, and mid‐IR spectroscopy to quantify non‐invasively biochemical markers via the dermal route, offering a convenient alternative to traditional invasive blood tests.

In the domain of personalized health, biophotonics facilitates the monitoring of a range of physiological parameters, including heart rate, oxygen saturation, and hydration levels. Such real‐time data facilitates the maintenance of optimal health and the early detection of potential health issues. The incorporation of AI into these sensors facilitates enhanced data analysis, thereby providing actionable insights and personalized health recommendations, which are also useful for all other unmet needs.

#### 2.3.7 New Applications: Agriculture and Food

The field of biophotonics plays a pivotal role in the advancement of agriculture, food quality, and safety. It offers a nondestructive, real‐time analysis of crops and food products, providing invaluable insights into their composition and quality. Techniques such as HSI and fluorescence spectroscopy are employed to monitor plant health, detect pathogens, and assess crop quality. Light‐based methods can also be employed for plant and growth control, reduction of herbicides, and treatment of fungi. These methods facilitate the optimization of agricultural practices, the reduction of food waste, the increase of durability, and the assurance of food safety and quality from the point of production to the point of consumption.

In the domain of food safety, biophotonic technologies are employed for the detection of contaminants, including pesticides, pathogens, toxins, and chemical or biological residues in food products. These techniques offer high sensitivity and specificity, thereby enabling the identification of trace amounts of harmful substances. By ensuring the safety and quality of food products, biophotonics contributes to public health and reduces the incidence of foodborne illnesses.

#### 2.3.8 New Applications: Environmental Monitoring

The field of environmental monitoring stands to gain considerably from the application of biophotonic technologies, which offer sophisticated methodologies for the detection and quantification of pollutants in air, water, and soil. Techniques such as Raman spectroscopy and plasmon‐enhanced fluorescence offer high sensitivity for the detection of contaminants at low concentrations. These methods are indispensable for the surveillance of environmental health and the assurance of compliance with regulatory standards.

In the context of water quality monitoring, biophotonics enables the detection of a range of pollutants, including heavy metals, pesticides, and microbial contaminants. These techniques provide data in real time, enabling prompt corrective action to avert environmental damage. Similarly, biophotonic sensors are employed for the monitoring of air quality, with the objective of detecting pollutants such as particulate matter and volatile organic compounds. In addition, light‐induced antimicrobial treatment can reduce contaminations (handrails, implants, contaminated surfaces, etc.). All this contributes to the creation of a healthier living environment.

#### 2.3.9 New Applications: Further Topics

Biophotonic capabilities are widely utilized in gene and protein sequencing technologies and are at the core of the rapidly expanding spatial proteomics sector, where omics analysis is applied to each pixel in a field of view, requiring advanced microscopy techniques. This offers great potential for understanding and diagnosing diseases. Biophotonic techniques are also essential for flow cytometry, increasingly enabling advances in the ability to identify and sort cells. Biophotonics may assist with label‐free diagnosis and ultimately selection of the embryo for enhancing the success rate of assisted reproductive technologies such as IVF.

Biophotonics presents a promising avenue for process analytical technology (PAT), offering the potential for real‐time monitoring of in‐line processes. This could facilitate prompt intervention in the event of process alterations that may compromise the quality of the end product.

In forensic science, biophotonic technologies offer noninvasive methods for analyzing biological samples, such as blood and tissue, without destroying evidence. Techniques like Raman spectroscopy can identify substances and determine their composition, aiding in criminal investigations.

Biophotonics also finds applications in veterinary medicine, where it is used for diagnostics and treatment of animal diseases. Techniques such as fluorescence imaging and laser therapy are employed for noninvasive diagnostics and targeted treatments, improving animal health and welfare. IR spectroscopy is already used for routine monitoring of milk, and there are many efforts underway to employ it routinely to monitor cow health.

#### 2.3.10 Technological Trends: Synergy With AI

All biophotonic investigations are underpinned by research into bespoke data evaluation algorithms, which facilitate the translation of experimental measurement data into information that is both qualitative and quantitative in nature. Data analysis is critical for any biophotonic approaches to be utilized in medical diagnostics or life and environmental science analysis. The latest methods of AI are becoming increasingly significant in this context. Modern machine learning methods, such as deep learning or classical AI methods, permit the extraction of greater information from photonic measurement data, including image data and spectral measurements, than is possible through manual assessment or classical chemical multicomponent analysis. The incorporation of AI into biophotonic imaging systems facilitates enhanced data interpretation, thereby enabling the formulation of personalized treatment plans based on individual patient profiles.

To date, the productive integration of AI and biophotonics is still in its infancy. There are significant synergies between optics/photonics and AI. First and foremost, the automated interpretation of large data sets with powerful AI methods, as opposed to the “naked eye,” opens entirely new avenues for the derivation of secondary data and conclusions from primary information. It is also noteworthy to mention the field of “Explainable AI,” which encompasses the investigation of visualization techniques that aim to transform the opaque nature of nonlinear AI methods into interpretable models. While conventional chemometrics and multivariate analysis based on latent‐variable methods are explainable AI techniques and still are useful, in particular for small data sets, it is increasingly realized that their intrinsic limitations, like the assumption of linearity, justify more sophisticated approaches.

Furthermore, virtual research environments and data‐sharing platforms can facilitate open science and accelerate progress in biophotonic research. Such platforms can facilitate convenient access to data sets, computational tools, and collaborative opportunities, thereby fostering a culture of shared knowledge and resources. They can assist with the training and education of students and the end‐users.

#### 2.3.11 Technological Trends: Combination With New Materials

The combination of photonics with new materials, such as metamaterials or photonic nanomaterials, can push biophotonic approaches to new levels of precision in terms of sensitivity, selectivity, or spatiotemporal resolution. The usage of plasmonic nanostructures offers new possibilities in terms of boosting linear and nonlinear Raman methods down to a single molecule level with the highest spectral and temporal resolution.

#### 2.3.12 Challenges and Future Directions

Despite the many benefits and applications of biophotonics, several challenges remain. The high cost of researching, developing, and deploying biophotonic technologies can limit their accessibility, particularly in resource‐constrained settings. Technical complexity and the need for specialized expertise can also hinder widespread adoption. Furthermore, navigating the regulatory landscape for new medical devices is often a lengthy and complex process, posing additional barriers to market entry. In this context, methods to compare biophotonic technologies and the development of references and standards need to be pursued.

To overcome these challenges, continued investment in research and development (R&D) is essential. Collaborative efforts between researchers, clinicians, industry stakeholders, and regulatory bodies can drive innovation and facilitate the translation of biophotonic technologies from the laboratory to clinical practice. Interdisciplinary education and training programs can help build the necessary expertise and ensure that healthcare professionals are equipped to utilize these advanced technologies effectively and reduce potential bias and prejudice. There is also considerable scope for open‐source approaches to disseminating cost‐effective modular tools that can empower scientists in lower resource settings, where the burden of disease is often the most severe.

In conclusion, biophotonics addresses critical unmet needs and offers innovative solutions across various fields. Its applications in medical diagnostics, treatment, continuous health monitoring, agriculture, food safety, and environmental monitoring highlight its transformative potential. By addressing current challenges and leveraging opportunities for innovation and collaboration, biophotonics can significantly enhance healthcare outcomes and contribute to a healthier, safer world entirely in line with the One Health concept, which recognizes that the health of humans, animals, and the environment are interconnected and promotes a collaborative, multisectoral approach to address health challenges at the interface of these domains. Continued R&D, coupled with interdisciplinary collaboration, will further enhance the capabilities and applications of biophotonics, driving its adoption and integration into various industries.

In the context of future directions, it is also noteworthy to mention the emerging field of quantum biophotonics, which holds immense promise for advancing our understanding of biological processes and developing new tools for biomedical applications. The exploitation of the quantum properties of nonclassical states of light enables the development of novel biophotonic imaging and sensing approaches, which have the potential to revolutionize biomedical diagnostics, facilitating the creation of images that transcend the limitations of current technology, for example, imaging below the shot noise limit and in low light conditions. The ability to quantify novel characteristics of a light field, extending beyond the conventional parameters of intensity distribution, offers a promising avenue for exploring innovative conceptual and technological avenues in the domain of optical health technologies. In this context, it is crucial to highlight the development of light sources capable of generating noise‐free quantum states of light, imaging techniques that rely on the correlations between different photons, and detector systems that are able to measure correlations in light to a degree that far exceeds the capabilities of classical cameras.

## 3 Translating Biophotonic Solutions Into Market‐Ready Applications and Products

The field of biophotonics holds immense promise for advancing healthcare. However, the journey from innovative research to market‐ready applications and products involves several challenges and complexities, which were discussed in three dedicated sessions at ICOB.

### 3.1 Understanding the Market Needs

The first step in translating biophotonic research into market‐ready solutions is to understand the unmet needs and demands of the market. This requires a thorough analysis of current applicational challenges, gaps in existing technologies, and the potential impact of biophotonic innovations. Researchers and developers must engage with healthcare providers, patients, and industry stakeholders to gather insights and validate the relevance of their solutions. The same applies to all other application areas where biophotonic technologies can make a difference.

### 3.2 Rigorous R&D

The advancement of biophotonic technologies necessitates a meticulous approach to R&D. This necessitates a substantial degree of experimentation, prototyping, validation, and the development of standards, SOPs, and so on to guarantee the technology's robustness, reliability, reproducibility, and efficacy. The primary objectives are to enhance the sensitivity and specificity of biophotonic devices, optimize imaging resolution, and guarantee the safety and efficacy of therapeutic applications. It is imperative that interdisciplinary teams comprising biologists, physicists, engineers, and people with domain expertise like clinicians collaborate to address the complex challenges inherent to R&D.

### 3.3 Regulatory Compliance and Approval

Navigating the regulatory landscape is one of the most significant challenges in bringing biophotonic solutions to the medical device market. Regulatory bodies such as the FDA in the United States and the EMA in Europe as well as EFSA in the food sector have stringent requirements to ensure the safety and efficacy of medical devices and technologies. Developers must conduct pre‐clinical and clinical trials to gather evidence supporting their product's claims. Evidence gathered by studies on each level is necessary. This process is time‐consuming and costly, but it is essential for obtaining the necessary approvals to market the product.

### 3.4 Intellectual Property (IP) and Patents

Protecting IP is necessary for commercial investment in biophotonic innovations. In many cases, IP cannot be kept secret, because backward engineering is possible. In such cases, patents provide legal protection against unauthorized use and help secure a competitive advantage in the market to justify the considerable investment required to obtain regulatory approval. The process of obtaining patents can be complex, requiring detailed documentation of the invention and a clear demonstration of its novelty and utility. Researchers and developers must work with IP experts to navigate the patent landscape, identify potential infringements, and secure robust patent protection.

### 3.5 Manufacturing and Scalability

Once a biophotonic solution has been validated and approved, the next step is to scale up manufacturing to meet market demand. This involves developing scalable production processes, sourcing high‐quality materials, and ensuring consistent quality control. Manufacturers must also consider the cost of production to ensure the product is economically viable. Advanced manufacturing techniques, such as photonic integrated circuits (PICs) and lab‐on‐a‐chip technologies, can help reduce costs and improve scalability.

### 3.6 Market Entry and Commercialization

Successfully bringing a biophotonic product to market requires a well‐defined commercialization strategy. This includes identifying target markets, developing marketing and sales plans, and establishing distribution channels. Companies must also engage in strategic partnerships with service providers, distributors, and other stakeholders to facilitate market entry and adoption. Effective communication of the product's value proposition, backed by clinical evidence, is essential to gain the trust and acceptance of end‐users. In addition, health insurance providers must be convinced that new medical devices should be covered for the end user. In clinics, efforts must be made to persuade decision‐makers to adopt new technologies (including new devices) for diagnosis and therapy and to ensure that clinics can bill for their use. Food producers must also be convinced that the implementation of biophotonic technologies is beneficial for them.

### 3.7 Education and Training

To ensure the successful adoption of biophotonic technologies, it is important to provide education and training to the domain professionals and students, for example, in medical faculties and nurse schools. This involves developing comprehensive training programs that cover the operation, maintenance, and application of biophotonic devices. Training sessions, workshops, and continuous professional development courses can help clinicians and technicians become proficient in using these advanced technologies, thereby improving patient outcomes.

### 3.8 Post‐Market Surveillance and Continuous Improvement

The journey does not end once a biophotonic product is on the market. Continuous monitoring and post‐market surveillance are essential to ensure the product's ongoing safety and effectiveness. Gathering feedback from users, monitoring for adverse events, and conducting periodic reviews are part of this process. Based on the feedback and data collected, companies can implement improvements and updates to enhance the product's performance and address any emerging issues.

### 3.9 Addressing Ethical and Societal Implications

The development and deployment of biophotonic technologies raise important ethical and societal questions. Issues related to patient privacy, data security, and the equitable distribution of advanced medical technologies must be carefully considered. Dependencies of unmet needs and procedures on culture and ethnicity must be taken into account. Developers must engage with ethicists, policymakers, and the public to address these concerns and ensure that the technologies are developed and used responsibly. Clear guidelines and transparent communication are essential to build public trust and acceptance.

### 3.10 Investment and Funding

Securing adequate funding is crucial at every stage of translating biophotonic research into market‐ready products. This includes funding for R&D, clinical trials, regulatory compliance, manufacturing, and marketing. Researchers and developers must explore various funding sources, including government grants, venture capital, and public‐private partnerships. Building a strong business case and demonstrating the potential market impact of the technology can help attract investors and secure the necessary financial support.

### 3.11 Interdisciplinary Collaboration and Networking

Successful translation of biophotonic solutions requires collaboration across multiple disciplines and sectors. Networking with academic institutions, industry partners, healthcare providers, and regulatory bodies can facilitate knowledge exchange, resource sharing, and collaborative problem‐solving. Attending conferences, participating in industry forums, and joining professional networks can help researchers and developers stay abreast of the latest advancements and forge valuable partnerships.

### 3.12 Case Studies and Best Practices

Examining successful case studies of biophotonic technologies that have made it to market can provide valuable insights and best practices. Analyzing the strategies and approaches used by companies that have successfully navigated the translation process can highlight key lessons and potential pitfalls. These case studies can serve as models for emerging developers and help them refine their strategies for bringing biophotonic solutions to market.

In conclusion, translating biophotonic solutions from research to market‐ready applications involves a multifaceted process that includes understanding market needs, rigorous R&D, regulatory compliance, IP protection, scalable manufacturing, and strategic commercialization. Addressing these challenges requires a coordinated effort across various disciplines and sectors, supported by robust funding and collaboration. By navigating these complexities effectively, biophotonic technologies can realize their full potential to revolutionize healthcare and improve patient outcomes.

## 4 Recommendations for Advancing the Development of Biophotonics

The field of photonic health technologies holds immense potential for revolutionizing medical diagnostics, treatment, and research. However, realizing this potential requires addressing several challenges and strategically advancing the development of biophotonics in all its various facets. The following presents a number of suggestions discussed in the ICOB sessions.

### 4.1 Investment in R&D

One of the fundamental steps to advancing biophotonics is sustained investment in R&D. This investment should focus on both basic and applied research to explore new applications of biophotonics and to refine existing technologies. Funding from government agencies, private sectors, and public‐private partnerships can significantly boost innovation in this field. Establishing dedicated research centers and laboratories equipped with state‐of‐the‐art technologies can provide the necessary infrastructure for groundbreaking discoveries.

Interdisciplinary collaboration is crucial for R&D in biophotonics. Bringing together experts from physics, biology, chemistry, engineering, medicine, food and agricultural as well as environmental sciences can foster a multidisciplinary approach to problem‐solving, leading to innovative solutions. Joint research initiatives and collaborative projects can facilitate the exchange of knowledge and resources, accelerating the development of new biophotonic techniques and applications.

### 4.2 Enhancing Education and Training

To support the growth of biophotonics, it is essential to develop comprehensive educational programs that provide specialized training in this field. Universities and research institutions could offer dedicated offline and online courses and degree programs in biophotonics, covering both theoretical and practical aspects. These programs should include hands‐on training with advanced biophotonic instruments, such as OCT, fluorescence imaging, and Raman spectroscopy.

Moreover, continuous professional development opportunities should be available for researchers and clinicians already working in the field. Workshops, seminars, and online courses can help professionals stay updated with the latest advancements in biophotonics and enhance their technical skills. Encouraging participation in international conferences and symposia can also facilitate knowledge sharing and networking among biophotonics experts.

### 4.3 Addressing Technical Challenges

Biophotonic technologies offer great potential to alleviate costs in the medical and other application fields by enabling more efficient and precise procedures. However, for clinics and laboratories to benefit from these advancements, they must be willing to invest in innovative biophotonic equipment and the staff to operate them. While initial investment is necessary, the long‐term benefits can outweigh these costs by improving, for example, patient outcomes and reducing the need for more expensive or invasive traditional treatments. A lack of investment funds could, however, slow the market penetration of these cutting‐edge solutions. Therefore, the focus should be on developing user‐friendly and accessible devices to encourage broader adoption.

Improving the standardization and calibration of biophotonic instruments is also critical. Consistency in performance and accuracy across different devices (or the same devices and different labs) and settings is essential for reliable diagnostics, continuous monitoring, and research outcomes. Establishing standardized protocols and guidelines for the use of biophotonic technologies can ensure uniformity and comparability of results, streamlined application, and higher use value.

### 4.4 Fostering Industry‐Academia Partnerships

Collaboration between academia and industry is vital for translating biophotonic research into market‐ready products. Industry partners can provide the necessary resources for scaling up production, conducting clinical trials, and navigating regulatory pathways. Academia, on the other hand, can contribute cutting‐edge research and innovative ideas.

Creating platforms for industry‐academia collaboration, such as innovation hubs and technology transfer offices, can facilitate the commercialization of biophotonic technologies. These platforms can support the entire innovation pipeline, from initial research to product development and market entry. By working together, academia and industry can accelerate the development of biophotonic solutions that meet clinical and market needs.

### 4.5 Navigating Regulatory and Ethical Challenges

The regulatory landscape for medical devices, including biophotonic and laser medical technologies, is complex and often poses significant challenges. To streamline the approval process, developers should engage with regulatory bodies early in the development cycle. Understanding the regulatory requirements and designing studies to meet these criteria can facilitate smoother and faster approval.

Addressing ethical considerations is also crucial, especially when dealing with patient data and AI‐driven biophotonic applications. Developing clear guidelines for ethical use and ensuring data privacy and security can build trust among stakeholders. Engaging with ethicists and regulatory experts can help navigate these challenges and ensure compliance with ethical standards.

### 4.6 Leveraging AI

As mentioned above, AI has the potential to significantly enhance the capabilities of biophotonic technologies. AI algorithms can analyze large data sets generated by biophotonic instruments, providing automated and accurate interpretation of complex spectral or imaging data. Integrating AI with biophotonics can improve diagnostic accuracy, facilitate real‐time decision‐making, and enable personalized treatment planning.

To leverage AI effectively, it is essential to develop robust and high‐quality data sets for training AI models. Collaboration with data scientists and AI experts can help optimize algorithms for biophotonic applications. Additionally, ensuring transparency and explainability of AI‐driven decisions is crucial for gaining clinical acceptance and trust.

### 4.7 Promoting Public Awareness and Engagement

Raising public awareness about the benefits and applications of biophotonics can drive demand and support for these technologies. Public engagement initiatives, such as educational campaigns, exhibitions, and interactive demonstrations, can inform and inspire the public about the potential of biophotonics.

Engaging with policymakers and healthcare as well as other domain‐related providers is also important to advocate for the integration of biophotonic technologies into the corresponding systems. Highlighting the end‐user and economic benefits of biophotonics can encourage investment and adoption at institutional and governmental levels.

### 4.8 Exploring New Applications

While biophotonics has already shown significant promise in medical diagnostics and laser medical treatment as well as in other application areas, there are numerous potential applications that remain underexplored. Expanding applications in these areas can have a substantial impact.

Investing in research to explore these new applications can open additional markets and broaden the impact of biophotonics. Collaborative projects with environmental agencies, agricultural organizations, and food safety regulators following the One Health concept can facilitate the development and deployment of biophotonic solutions in these fields.

### 4.9 Encouraging Innovation Through Funding and Incentives

Providing financial incentives and funding opportunities for biophotonic R&D can stimulate innovation. Grants, tax credits, and subsidies for biophotonic projects can encourage researchers and companies to invest in this field. Funding programs should support both fundamental research and applied projects aimed at developing market‐ready products.

Governments and funding agencies can also create innovation challenges and competitions to encourage creative solutions and foster a competitive but complementary and cooperative spirit among researchers and developers. Recognizing and rewarding successful innovations can further motivate the biophotonic community to push the boundaries of what is possible.

Advancing the development of biophotonics requires a multifaceted approach that addresses technical, educational, regulatory, and financial challenges. By investing in R&D, enhancing education and training, fostering industry‐academia partnerships, and leveraging AI, the field of biophotonics can realize its full potential. Navigating regulatory and ethical challenges, promoting public awareness, exploring new applications, and encouraging innovation through funding and incentives are also critical steps in this journey. Through collaborative efforts and strategic actions, biophotonics can transform healthcare and other fields, leading to improved outcomes and a brighter future.

**FIGURE 6:**
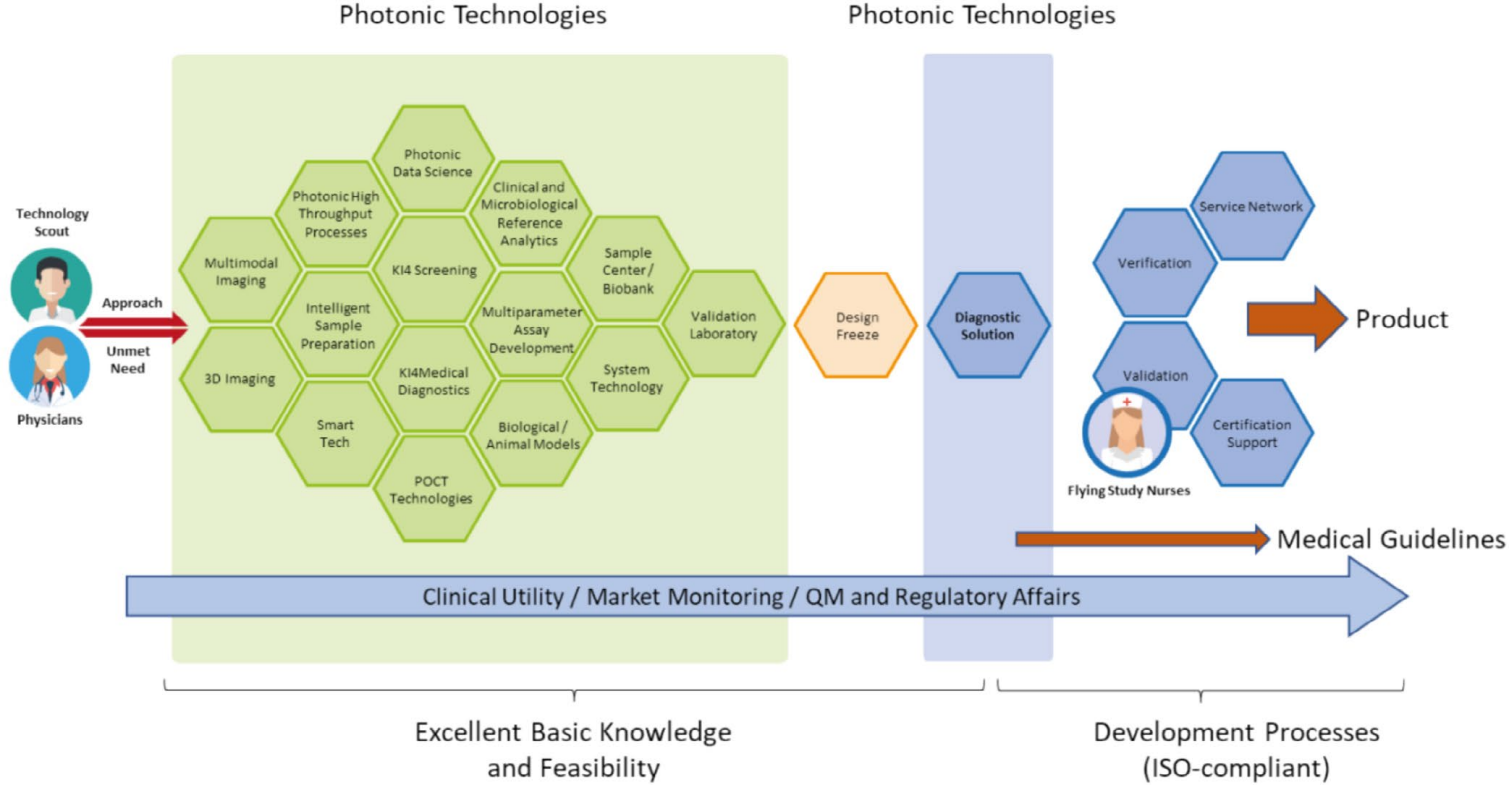
Workflow planned for the Leibniz Center for Photonics in Infection Research.

The Leibniz Center for Photonics in Infection Research (LPI) (see https://lpi‐jena.de/en/), which was recently included in the German government's national roadmap, is an example of how these recommendations can be successfully implemented. In the context of the LPI, the integration of photonic methods into infection research will facilitate the emergence of novel diagnostic and targeted therapeutic light‐based approaches. Following the necessary approvals, these will be transferred directly to industrial production and clinical application. A key component of the LPI is the Technology Scout, who works with clinicians at the beginning of the value chain to identify optimal photonic solutions that have demonstrated efficacy in proof‐of‐concept studies for specific medical problems. The LPI can thus serve as a model for other medical problems, such as cancer or neurodegenerative diseases, with the goal of overcoming the “valley of death” of clinical translation in these areas as well. Certainly, other examples of exemplary institutions exist and could be mentioned as well (Figure 6).

## 5 Summary: SWOT Analysis

As can be seen above and impressively witnessed during the ICOB 2024 conference, biophotonics has emerged as a powerful new scientific discipline with enormous application potential, particularly in medical diagnostics and laser treatment. However, there are still several hurdles to overcome before this potential fully reaches the patient or end‐user, as discussed at the conference and described above.

Ongoing advances in biophotonic technology research, such as the development of new, powerful hardware and software components, are opening up entirely new biophotonic applications, which have also been highlighted in this article. The following is a brief summary in the form of a SWOT analysis. Given the breadth of the field, we will not provide a detailed analysis of each application or technique. Instead, we will use examples to illustrate general principles. As a result, not all points will apply to every aspect of the field.

### 5.1 Strengths

#### 5.1.1 Precision and Non‐Invasiveness

Biophotonics and laser medicine offer highly precise imaging and diagnostic capabilities without the need for invasive procedures, reducing patient risk and discomfort.

#### 5.1.2 Real‐Time Diagnostic Capabilities

Biophotonic technologies incl. AI provides real‐time imaging, allowing for immediate and accurate decisions in all application fields and domains.

#### 5.1.3 Versatility in Applications

Biophotonics is applicable in various fields, including medical diagnostics, therapeutic interventions, environmental monitoring, food, and agriculture.

#### 5.1.4 Sensitivity and Specificity

Biophotonics offers high sensitivity and specificity, enabling the detection of minute changes in biological tissues, crucial for early diagnosis and monitoring.

#### 5.1.5 Time Resolution

Biophotonics can be applied to dynamic biological processes over a time scale from minutes to femtoseconds.

#### 5.1.6 Integration With AI

AI integration enhances diagnostic accuracy and data interpretation, facilitating personalized treatment planning.

#### 5.1.7 Accessibility and Portability

Advances in portable and cost‐effective diagnostic devices increase accessibility, particularly in remote or resource‐limited settings.

#### 5.1.8 Contribution to Fundamental Research

Biophotonics significantly advances biological and medical research by enabling detailed study of cellular and molecular processes.

#### 5.1.9 Economic and Clinical Impact

Early and accurate diagnosis through biophotonics reduces healthcare costs, reduces or minimizes recovery times, and improves patient outcomes.

### 5.2 Weaknesses

#### 5.2.1 High Costs for R&D

The development and advancement of new biophotonic technologies can require significant investment in research and innovation.

#### 5.2.2 Investment Costs

Clinics and medical laboratories need to be prepared to invest in cutting‐edge biophotonic equipment and appropriate staff. Limited funding or institutional support for such investments could slow the widespread adoption of these technologies.

#### 5.2.3 Technical Complexity

The operation and interpretation of biophotonic systems may require specialized knowledge, potentially posing a potential barrier to widespread adoption.

#### 5.2.4 Limited Standardization

Variability in device performance and lack of standardized calibration protocols can lead to inconsistent data, affecting reliability.

#### 5.2.5 Regulatory and Ethical Challenges

Navigating the complex regulatory landscape and addressing ethical concerns related to patient consent and data privacy are significant hurdles.

#### 5.2.6 Data Management Challenges

Managing and interpreting the large data sets generated by biophotonics, especially when integrated with AI, can be labor‐intensive and complex.

#### 5.2.7 Limited Field Deployment

Despite its potential, many biophotonic techniques remain confined to research laboratories and have not yet achieved widespread clinical adoption.

### 5.3 Opportunities

#### 5.3.1 Early Disease Detection

Biophotonic techniques offer noninvasive methods for the early detection of diseases and disease monitoring, particularly in oncology, cardiovascular health, infectious diseases, transplant medicine, and neuroscience.

#### 5.3.2 Faster Drug Discovery

Biophotonics‐based assay technologies are addressing challenges associated with drug resistance and relapse by studying heterogeneity in single‐cell‐resolved responses to therapies.

#### 5.3.3 Personalized Medicine

Biophotonic tools can be used for real‐time monitoring of patient responses to treatments, enabling the development of personalized therapeutic approaches, or for real‐time health monitoring to improve chronic disease management.

#### 5.3.4 Technological Advancements

Technological advancements (new detector or laser concepts, combination with nanotechnology, utilization of quantum imaging concepts, etc.) can lead to the development of highly sensitive sensor and imaging techniques, expanding the capabilities of current biophotonic tools in terms of sensitivity, resolution, and so on.

#### 5.3.5 AI and Machine Learning

AI and machine learning can be leveraged to analyze large data sets generated by biophotonic imaging techniques, improving diagnostic accuracy and treatment outcomes.

#### 5.3.6 Growing Market Demand due to

- *Aging Population*: As the global population ages, the demand for advanced diagnostic and therapeutic technologies is increasing, providing a robust market for biophotonic applications.
- *Emerging Markets*: There is growing interest and investment in biophotonics in emerging markets, particularly in Asia and Latin America, where healthcare infrastructure is rapidly developing.

#### 5.3.7 Environmental and Industrial Applications

Biophotonic techniques can be applied to monitor environmental pollutants, water quality, and food safety, offering opportunities in the environmental sector.

#### 5.3.8 Agricultural Technology

In agriculture, biophotonics can be used for plant health monitoring, pest detection, but also reducing herbicides in plants and antibiotics in animals and optimizing crop yields, which is crucial for addressing global food security challenges.

#### 5.3.9 Collaboration and Funding Opportunities

- *Government and Academic Partnerships*: Increased funding from government bodies and collaborations with academic institutions are fostering innovation and the development of new biophotonic technologies.
- *Public–Private Partnerships*: Collaborations between industry and research organizations can accelerate the commercialization of biophotonic innovations.
- *Venture Capital Interest*: Growing interest from venture capital firms in funding biophotonic startups can drive innovation and bring new products to market more quickly.

### 5.4 Threats

#### 5.4.1 Technical Challenges

The complexity of maintaining biophotonic systems, coupled with the need for specialized expertise, could hinder widespread adoption.

#### 5.4.2 Economic Barriers

High costs of development, maintenance, and competition in the medical technology market could limit accessibility and innovation.

#### 5.4.3 Regulatory Hurdles

The stringent and varied regulatory requirements across regions could delay the introduction of biophotonic innovations into the market.

#### 5.4.4 Ethical and Social Concerns

Issues like patient consent, data privacy, and the “black‐box” nature of AI could raise ethical concerns and limit trust in biophotonic technologies.

#### 5.4.5 Limited Practical Applications

**FIGURE 7:**
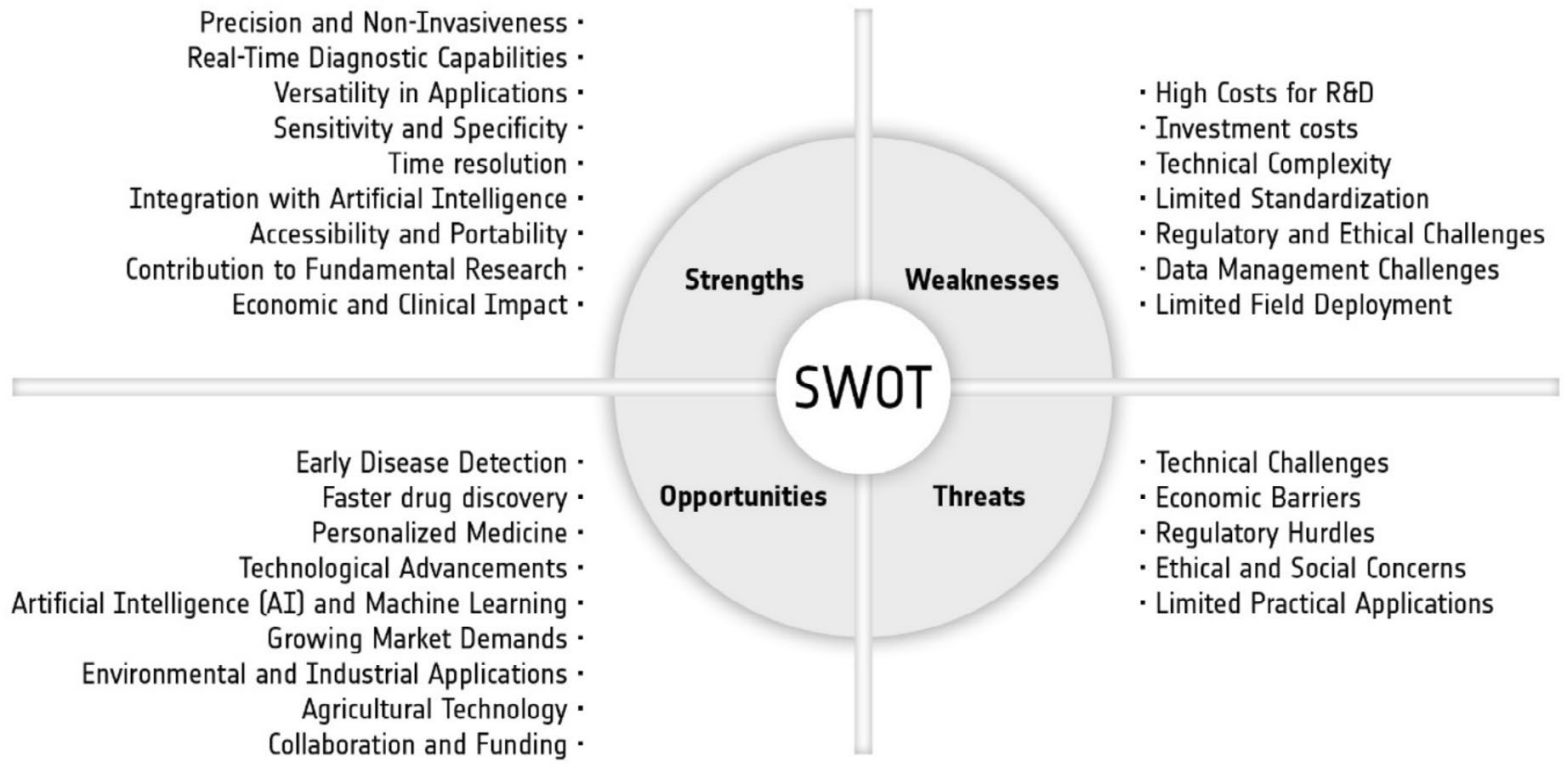
Summary and SWOT analysis.

Many biophotonic techniques are still in the research stages and have yet to be widely adopted in clinical settings, impeding their full potential (Figure 7).

## 6 Conclusion: The Transformative Potential and Future of Biophotonics and Laser Medicine

The exploration of biophotonics and its potential applications demonstrates significant advances and future prospects within this interdisciplinary field. As we examine the multifaceted world of biophotonics, it is essential to synthesize the critical insights and lessons derived from the ICOB sessions and the comprehensive analysis presented.

Biophotonics stands at the crossroads of biology, medicine, and photonics, leveraging light‐based technologies to investigate and manipulate biological systems. This inherent interdisciplinarity of this field draws from physics, chemistry, biology, engineering, and medicine, fostering a robust platform for innovation. The applications of biophotonics are vast and varied, spanning from medical diagnostics and therapeutic interventions to environmental monitoring and food safety. Already biophotonics has made major worldwide inroads to support human health: clear examples might include the pulse oximeter, OCT scans for eye health, and so on.

In the realm of oncology, biophotonics has the potential to revolutionize cancer detection and treatment. Techniques such as OCT, FLIM, and Raman spectroscopy‐based approaches offer unprecedented resolution and specificity, enabling early detection of malignancies and precise surgical guidance. PDT exemplifies the therapeutic potential of biophotonics, providing targeted treatment that minimizes damage to healthy tissues. Although only OCT is in widespread clinical use, these advancements underscore the field's potential to enhance clinical outcomes and reduce healthcare costs through noninvasive, highly accurate diagnostic and therapeutic tools.

The impact of biophotonics can extend to infectious diseases, where rapid and accurate diagnostic methods are paramount. Traditional culture techniques are often time‐consuming and lack sensitivity, whereas biophotonic technologies offer swifter pathogen identification and resistance profiling. The development of portable, point‐of‐care diagnostic devices further exemplifies the practical benefits of biophotonics, enabling timely and effective treatment decisions in clinical settings. These innovations are particularly crucial in managing outbreaks and preventing the spread of infectious diseases.

Cardiovascular diseases also benefit significantly from biophotonic innovations. Techniques such as OCT and PAI facilitate noninvasive imaging of blood vessels, aiding in the early detection of atherosclerosis and other vascular conditions. The precision of these technologies can enhance surgical interventions and improve patient outcomes. Moreover, the integration of AI with biophotonic imaging systems can enhance data analysis, to enable personalized treatment plans and continuous health monitoring.

Neurodegenerative diseases present unique challenges that biophotonics is well‐equipped to address. High‐resolution imaging techniques like two‐photon fluorescence microscopy and light‐sheet microscopy allow researchers to study neuronal structures and functions in great detail. Optogenetics enables precise control of neural activity, advancing our understanding of brain disorders and potential therapeutic targets. These capabilities position biophotonics as a critical contributor to neurological research and treatment.

Beyond healthcare, biophotonics plays a pivotal role in environmental monitoring and food safety. Techniques such as HSI and fluorescence spectroscopy provide real‐time, nondestructive analysis of crops and food products, ensuring quality and safety from farm to table. In environmental monitoring, biophotonic technologies detect pollutants and contaminants with high sensitivity, contributing to sustainable environmental practices and public health protection.

The field of biophotonics presents certain challenges, particularly in the R&D of new technologies, which often require substantial investment. For small and medium‐sized enterprises as well as startups, these costs can be a significant hurdle, potentially delaying the development of market‐ready products. Additionally, technical complexity and the need for specialized expertise may add to the difficulty. Regulatory approval processes for new medical technologies are tedious and also take time. Nevertheless, with strategic investments, interdisciplinary collaboration, and more efficient regulatory procedures, the potential for broader adoption and integration of biophotonic technologies into clinical practice remains promising.

Educational initiatives are paramount in advancing the field of biophotonics. Developing comprehensive training programs that encompass theoretical knowledge and practical skills is essential for cultivating the next generation of biophotonic experts. Continuous professional development opportunities and international collaboration can further enhance the expertise and innovation within this field.

In conclusion, biophotonics represents a transformative force in modern science and medicine. Its applications in oncology, infectious diseases, cardiovascular health, neurodegenerative disorders, therapeutic drug monitoring, environmental monitoring, and food safety underscore its vast potential. Addressing the current challenges through strategic investments, regulatory engagement, and educational initiatives will be crucial in harnessing the full capabilities of biophotonics. As we continue to explore and innovate within this field, biophotonics promises to revolutionize diagnostics, treatment, and research, ultimately improving health outcomes and contributing to a safer, healthier world.

## Conflicts of Interest

The authors declare no conflicts of interest.

## Data Availability

The data that support the findings of this study are available on request from the corresponding author. The data are not publicly available due to privacy or ethical restrictions.
